# Phenolic Profile and Bioactivities of *Sideritis perfoliata* L.: The Plant, Its Most Active Extract, and Its Broad Biological Properties

**DOI:** 10.3389/fphar.2019.01642

**Published:** 2020-02-14

**Authors:** Cengiz Sarikurkcu, Marcello Locatelli, Andrei Mocan, Gokhan Zengin, Bulent Kirkan

**Affiliations:** ^1^ Department of Analytical Chemistry, Afyonkarahisar University of Health Sciences, Faculty of Pharmacy, Afyonkarahisar, Turkey; ^2^ Department of Pharmacy, D'Annunzio University of Chieti—Pescara, Chieti, Italy; ^3^ Department of Pharmaceutical Botany, Iuliu Haţieganu University of Medicine and Pharmacy, Cluj-Napoca, Romania; ^4^ Department of Biology, Selcuk University, Science Faculty, Konya, Turkey; ^5^ Water Institute, Süleyman Demirel University, Isparta, Turkey

**Keywords:** *Sideritis perfoliata*, LC–ESI–MS/MS, phenolic profile, enzyme inhibitory activities, antioxidant activities

## Abstract

*Sideritis*, also named “ironwort,” “mountain tea,” or “shepherd's tea,” is a genus of flowering plants used as herbal medicine in traditional Mediterranean-area medicine systems, and these plants are generally consumed as a herbal tea. Its use as herbal tea and in traditional herbal medicine is quite popular. There are currently few studies on *Sideritis perfoliata* L., and only one reports the use of a liquid chromatography coupled to diode array detection and electrospray ionization tandem mass spectrometry (LC-DAD-ESI-MS^n^) profile and the content of phenolic compounds without considering a possible correlation with its biological activities. This paper aims to investigate the antioxidant activities by means of several different biological/biochemical assays (radical scavenging, reducing power, ferrous ion chelating, and total antioxidant by phosphomolybdenum and β-carotene bleaching methods) as well as analyze the enzyme inhibitory activities (against AChE (acetylcholinesterase), BChE (butyrylcholinesterase), tyrosinase, α-glucosidase, and α-amylase) as well as the total phenolics, flavonoids, and condensed tannins. The reported results on *Sideritis perfoliata* highlighted that methanol and water extracts generally showed higher radical scavenging and reducing power activities. A similar trend could be observed for phosphomolybdenum and ferrous ion chelating activities. Methanol extracts showed lower activity only for the β-carotene bleaching assay.

## Introduction

In recent years, natural products have gained momentum in the designing of novel pharmaceutical products and herbal drugs. Many natural products or herbal preparations are currently being used for the treatment of several ailments both in modern or traditional medicine (Shen, 2015; Boy et al., 2018).

*Sideritis* (Lamiaceae), also named “ironwort,” “mountain tea,” or “shepherd's tea,” is a genus of flowering plants used as herbal medicine; it has several traditional uses, and it is generally consumed as herbal tea. The genus contains about 150 species, and these are distributed in the temperate climate of the Northern hemisphere (González-Burgos et al., 2011). The plants appear as short (8–50 cm) xerophytic subshrubs or herbs, annual or perennial, that grow at high altitudes (generally over 1000 m) with little or no soil and often on the surface of rocks. This genus is widely distributed in several Mediterranean regions, in the Balkans, in the Iberian Peninsula, in the Macaronesia, in Central Europe, and in temperate regions of Asia (Barber, 2000; Lindqvist and Albert, 2002).

Its use as herbal tea and in traditional herbal medicine is popular in Greece, Turkey, Albania, Kosovo, Bulgaria, and the Republic of Macedonia. Particularly, *Sideritis scardica* Griseb., used as herbal tea, is frequently prepared by decoction or infusion of the stems, leaves, and flowers (aerial parts). This herbal medicine is used traditionally to aid digestion, enforce the immune system, and reduce the symptoms of various illnesses. Several studies have reported a possible correlation with a positive effect of this plant on many common ailments (González-Burgos et al., 2011; Tadić et al., 2012), particularly with related to its anti-microbial, anti-inflammatory, and anti-oxidant activities. The main active compounds present in this genus are diterpenoids and flavonoids (Villar et al., 1986).

*S. perfoliata* is perennial with simple or branched stems and grows to height of 60 cm. Leaves are ovate, oblong, or oblong-lanceolate. Flowers are yellow in color. The species is mainly distributed to the east and Mediterranean region of Turkey (Davis, 1982). *S. perfoliata* is of great interest in term of traditional uses as it has antimicrobial, analgesic, stomachic, and carminative properties (Gonzalez-Burgos et al., 2011; Lall et al., 2019). There are only a few papers written on *Sideritis perfoliata* L. (Loizzo et al., 2007; Charami et al., 2008; Loizzo et al., 2008; Petreska et al., 2011; Çelik et al., 2016), and only one reports its LC-DAD-ESI-MS^n^ profile and content of phenolic compounds (3 hydroxycinnamic acid derivatives, 8 phenylethanoid glycosides, and 24 flavonoid glycosides) (Petreska et al., 2011). The other works report the biological activities merely against angiotensin converting enzyme (ACE), digestive enzymes related to diabetes (Loizzo et al., 2008), and the antioxidant and anti-inflammatory (Charami et al., 2008) as well as cytotoxic activity of essential oils (Loizzo et al., 2007). In addition, *S. perfoliata* is widely used in traditional medicine as an anti-inflammatory, anti-ulcer, vasoprotector, and for treatment against colds, coughs, and the flu.

Following our previous research on plant materials used as tea (Melucci et al., 2013), foods (Sobolev et al., 2018; Di Sotto et al., 2018), food supplements (Locatelli et al., 2017), traditional herbal medicine (Uysal et al., 2017), and their content of toxic/heavy metals (Locatelli et al., 2014; Melucci et al., 2018), we highlight and complete here the phytochemical information on this traditionally used medicinal plant.

In current research, we aimed to determine the antioxidant capacity of three extracts (ethyl acetate, methanol, and water) from *Sideritis perfoliata* by means of several chemical assays (radical scavenging, reducing power, ferrous ion chelating, and total antioxidant by phosphomolybdenum and β-carotene bleaching methods) and to explain their inhibitory effects against AChE, BchE, tyrosinase, α-glucosidase, and α-amylase. In addition, the chemical characterization for these extracts were performed by spectrophotometric and chromatographic methods.

## Materials and Methods

### Chemicals

Gallic acid, (+)-catechin, pyrocatechol, chlorogenic acid, 2,5-dihydroxybenzoic acid, 4-hydroxybenzoic acid, (−)-epicatechin, caffeic acid, syringic acid, vanillin, taxifolin, sinapic acid, *p*-coumaric acid, ferulic acid, rosmarinic acid, 2-hydroxycinnamic acid, pinoresinol, quercetin, luteolin, and apigenin were purchased from Sigma-Aldrich (St. Louis, MO, USA). Vanillic acid, 3-hydroxybenzoic acid, 3,4-dihydroxyphenylacetic acid, apigenin 7-glucoside, luteolin 7-glucoside, hesperidin, eriodictyol, and kaempferol were obtained from Fluka (St. Louis, MO, USA). Finally, verbascoside, protocatechuic acid, and hyperoside were purchased from HWI Analytik (Ruelzheim, Germany). Methanol and formic acid of HPLC grade were purchased from Sigma-Aldrich (St. Louis, MO, USA) and Merck (Darmstadt, Germany), respectively. Ultra-pure water (18 mΩ) was obtained from a Milli-Q water purification system (Millipore Co., Ltd.)

Ethyl acetate and methanol were obtained from Carlo Erba Reagents (Milan, Italy). Ultra-pure water was obtained using a Millipore Milli-Q Plus water treatment system (Millipore Bedford Corp., Bedford, MA). All chemicals were of analytical grade.

### Plant Material

Aerial parts of *Sideritis perfoliata* L. were purchased from the local market in Isparta Province in the Mediterranean Region of Turkey on 15 January 2014 (1020 m, 37° 45' 52.90”N 30° 33' 16.02”E). The plant was authenticated by Dr. Olcay Ceylan and deposited at the herbarium of the Department of Biology, Mugla Sitki Kocman University (Mugla-Turkey) under the accession no. OC.3003.

### Preparation of the Extracts

Air-dried aerial parts of *S. perfoliata* were individually extracted by using a Soxhlet extractor for 5 h with 250 mL of ethyl acetate, methanol, or water as previously described by Sarikurkcu et al. (2014). All extracts were stored at +4°C until the analyses, and all analysis were completed in 10 days.

### Total Bioactive Components

Total phenolic, flavonoid, and condensed tannin content was determined by employing the methods given in the literature (Zengin et al., 2014). For total phenolic content, the sample solution (0.25 mL) was mixed with diluted Folin–Ciocalteu reagent (1 mL, 1:9) and shaken vigorously. After 3 min, a Na_2_CO_3_ solution (0.75 mL, 1%) was added, and the sample absorbance was read at 760 nm after 2 h incubation at room temperature. Total phenolic content was expressed as equivalents of gallic acid.

For total flavonoid content, the sample solution (1 mL) was mixed with the same volume of aluminium trichloride (2%) in methanol. Similarly, a blank was prepared by adding the sample solution (1 mL) to methanol (1 mL) without AlCl_3_. The sample and blank absorbance were read at 415 nm after 10 min incubation at room temperature. Absorbance of the blank was subtracted from that of the sample. Total flavonoid content was expressed as equivalents of quercetin.

For total condensed tannin content, sample solution (0.5 mL), was mixed with a vanillin reagent [1.5 mL, 1% in 7 M H_2_SO_4_ (96%)] in an ice bath and then mixed well. Similarly, a blank was prepared by adding the sample solution (0.5 mL) to 7 M H_2_SO_4_ (1.5 mL). The sample and blank absorbances were read at 500 nm after a 15 min incubation at room temperature. The absorbance of the blank was subtracted from that of the sample. The total condensed tannin content was expressed as equivalents of (+)-catechin.

### Liquid Chromatography-Electrospray Ionization-Tandem Mass Spectrometry (LC–ESI–MS/MS) Analysis

An Agilent Technologies 1260 Infinity liquid chromatography system hyphenated to a 6420 Triple Quad mass spectrometer was used for quantitative analyses. Chromatographic separation was carried out on a Poroshell 120 EC-C18 (100 mm × 4.6 mm I.D., 2.7 μm) column. Three mobile phases were tested to obtain a complete resolution of all isomers and the highest sensitivity for all target compounds, namely (i) 0.1% formic acid/methanol, (ii) 5 mM ammonium acetate/acetonitrile with 0.1% acetic acid, and (iii) 10 mM ammonium formate with 0.1% formic acid/acetonitrile with 0.1% formic acid. The first mobile phase configuration (0.1% formic acid/methanol) was selected on the base of the better chromatographic resolution of isomeric compounds. On the other hand, the selected mobile phase configuration also provided higher sensitivity for most of the phenolic compounds.

As a result, the mobile phase was made up from solvent A (0.1%, *v*/*v* formic acid solution) and solvent B (methanol). The gradient profile was set: 0.0 min 2% B eluent, 3.0 min 2% B eluent, 6.0 min 25% B eluent, 10.0 min 50% B eluent, 14.0 min 95% B eluent, 17.0 min 95% B, and 17.5 min 2% B eluent. The column temperature was thermostated at 25°C. The flow rate was 0.4 mL/min, and the injection volume was 2.0 μL (Cittan and Çelik, 2018).

The tandem mass spectrometer was coupled to the LC system using an ESI source. The electrospray source of the MS was operated in a negative and positive ionization mode, and the data acquisition was conducted in multiple reaction monitoring (MRM). The ESI source interface conditions were set: capillary voltage was −3.5 kV, gas temperature was 300°C, and gas flow was 11 L min^−1^. The nebulizer pressure was 40 psi. Analytical characters are given in **Table 1**.

**Table 1:** ESI–MS/MS Parameters and analytical characteristics for the Analysis of Target Analytes by MRM Negative and Positive Ionization Mode.

Target compounds	Rt (min)	Precursor ion	MRM1 (CE, V)	MRM2 (CE, V)	Linear equation	R^2^	LOD (μg/L)	LOQ (μg/L)
*Compounds analyzed by NI mode*								
Gallic acid	8.891	168.9 [M − H]−	125.0 (10)	–	y = 4.82x−26.48	0.9988	1.46	4.88
Protocatechuic acid	10.818	152.9 [M − H]−	108.9 (12)	–	y = 5.65x−9.99	0.9990	1.17	3.88
3,4-Dihydroxyphenylacetic acid	11.224	167.0 [M − H]−	123.0 (2)	–	y = 5.13x−12.39	0.9990	1.35	4.51
(+)-Catechin	11.369	289.0 [M − H]−	245.0 (6)	202.9 (12)	y = 1.45x+1.95	0.9974	3.96	13.20
Pyrocatechol	11.506	109.0 [M − H]−	90.6 (18)	52.9 (16)	y = 0.11x−0.52	0.9916	9.62	32.08
2,5-Dihydroxybenzoic acid	12.412	152.9 [M − H]−	109.0 (10)	–	y = 3.79x−14.12	0.9980	2.12	7.08
4-Hydroxybenzoic acid	12.439	136.9 [M − H]−	93.1 (14)	–	y = 7.62x+22.79	0.9996	1.72	5.72
Caffeic acid	12.841	179.0 [M − H]−	135.0 (12)	–	y = 11.09x+16.73	0.9997	3.15	10.50
Vanillic acid	12.843	166.9 [M − H]−	151.8 (10)	122.6 (6)	y = 0.49x−1.61	0.9968	2.56	8.54
Syringic acid	12.963	196.9 [M − H]−	181.9 (8)	152.8 (6)	y = 0.74x−1.54	0.9975	3.75	12.50
3-Hydroxybenzoic acid	13.259	137.0 [M − H]−	93.0 (6)	–	y = 3.69x−12.29	0.9991	1.86	6.20
Vanillin	13.397	151.0 [M − H]−	136.0 (10)	–	y = 2.02x+135.49	0.9926	15.23	50.77
Verbascoside	13.589	623.0 [M − H]−	461.0 (26)	160.8 (36)	y = 8.59x−28.05	0.9988	0.82	2.75
Taxifolin	13.909	303.0 [M − H]−	285.1 (2)	125.0 (14)	y = 12.32x+9.98	0.9993	1.82	6.05
Sinapic acid	13.992	222.9 [M − H]−	207.9 (6)	163.8 (6)	y = 2.09x−6.79	0.9974	2.64	8.78
p-Coumaric acid	14.022	162.9 [M − H]−	119.0 (12)	–	y = 17.51x+53.73	0.9997	1.93	6.44
Ferulic acid	14.120	193.0 [M − H]−	177.8 (8)	134.0 (12)	y = 3.32x−4.30	0.9992	1.43	4.76
Luteolin 7-glucoside	14.266	447.1 [M − H]−	285.0 (24)	–	y = 45.25x+156.48	0.9996	0.45	1.51
Rosmarinic acid	14.600	359.0 [M − H]−	196.9 (10)	160.9 (10)	y = 9.82x−17.98	0.9989	0.57	1.89
2-Hydroxycinnamic acid	15.031	162.9 [M − H]−	119.1 (10)	–	y = 16.72x−26.94	0.9996	0.61	2.03
Pinoresinol	15.118	357.0 [M − H]−	151.0 (12)	135.7 (34)	y = 0.80x−2.69	0.9966	3.94	13.12
Eriodictyol	15.247	287.0 [M − H]−	151.0 (4)	134.9 (22)	y = 14.24x−0.50	0.9998	0.80	2.68
Quercetin	15.668	301.0 [M − H]−	178.6 (10)	151.0 (16)	y = 14.68x−18.25	0.9997	1.23	4.10
Kaempferol	16.236	285.0 [M − H]−	242.8 (16)	229.1 (18)	y = 0.82x−3.06	0.9959	3.30	10.99
*Compounds analyzed by PI mode*								
Chlorogenic acid	11.802	355.0 [M + H]+	163.0 (10)	–	y = 12.14x+32.34	0.9995	0.55	1.82
(−)-Epicatechin	12.458	291.0 [M + H]+	139.1 (12)	122.9 (36)	y = 9.11x−9.99	0.9971	1.85	6.18
Hesperidin	14.412	611.1 [M + H]+	449.2 (4)	303.0 (20)	y = 5.98x+0.42	0.9993	1.73	5.77
Hyperoside	14.506	465.1 [M + H]+	303.1 (8)	–	y = 16.32x−1.26	0.9998	0.99	3.31
Apigenin 7-glucoside	14.781	433.1 [M + H]+	271.0 (18)	–	y = 21.33x−31.69	0.9983	0.41	1.35
Luteolin	15.923	287.0 [M + H]+	153.1 (34)	135.1 (36)	y = 8.96x+26.80	0.9992	1.34	4.46
Apigenin	16.382	271.0 [M + H]+	153.0 (34)	119.1 (36)	y = 11.29x+38.05	0.9987	0.96	3.20

R_t_, retention time; NI, negative ion; and PI, positive ion.

### Antioxidant Activity

Antioxidant activities of *Sideritis perfoliata* extracts were investigated by using several assays: total antioxidant capacity (by phosphomolybdenum and β-carotene-linoleic acid), ferrous ion chelating, reducing power [cupric ion reducing (CUPRAC), ferric reducing antioxidant power (FRAP), and potassium ferricyanide], and free radical scavenging [on 1,1-diphenyl-2-picrylhydrazyl (DPPH^·^) radical, 2,2-azino-bis (3-ethylbenzothiazloine-6-sulphonic acid) radical cation (ABTS^·+^), superoxide anion (O_2_^·-^) radical, and nitric oxide (^·^NO) radical]. All of the analyses were carried out by using the experimental conditions reported by Zengin et al. (2015).

Total antioxidant activity by β-carotene–linoleic acid method is determined by measuring the inhibition of the conjugated diene hydroperoxides arising from linoleic acid oxidation. A stock solution of β-carotene–linoleic acid mixture was prepared: 0.5 mg β-carotene was dissolved in chloroform (1 mL, HPLC grade). 25 μL linoleic acid and 200 mg Tween 40 was added. Chloroform was completely evaporated using a vacuum evaporator. Then 100 mL of oxygenated distilled water was added with vigorous shaking; 1.5 mL of this reaction mixture was dispersed to test tubes, sample solutions (0.50 mL, 2 mg/mL) were added, and the emulsion system was incubated for up to 2 h at 50°C. The same procedure was repeated with the standards and a blank. After this incubation period, the sample absorbance was read at 490 nm. Measurement of absorbance was continued until the color of β-carotene disappeared.

For total antioxidant activity by phosphomolybdenum method, the sample solution (0.3 mL) was combined with 3 mL of reagent solution (0.6 M sulfuric acid (96%), 28 mM sodium phosphate, and 4 mM ammonium molybdate). The sample absorbance was read at 695 nm after 90 min incubation at 95°C. The total antioxidant capacity was expressed as equivalents of ascorbic acid.

For ferrous ion chelating activity, the sample solution (2 mL) was added to FeCl_2_ solution (0.05 mL, 2 mM). The reaction was initiated by the addition of 5 mM ferrozine (1 mM, 0.2 mL). Similarly, a blank was prepared by adding the sample solution (2 mL) to FeCl_2_ solution (0.05 mL, 2 mM) and water (0.2 mL) without ferrozine. Then, the sample and blank absorbances were read at 562 nm after 10 min incubation at room temperature. The absorbance of the blank was subtracted from that of the sample. Ferrous ion chelating activity was expressed as equivalents of EDTA (ethylenediaminetetraacetic acid disodium salt).

For CUPRAC reducing power, the sample solution (0.5 mL) was added to a premixed reaction mixture containing CuCl_2_ (1 mL, 10 mM), neocuproine (1 mL, 7.5 mM), and NH4Ac buffer (1 mL, 1 M, pH 7.0). Similarly, a blank was prepared by adding the sample solution (0.5 mL) to the premixed reaction mixture (3 mL) without CuCl_2_. Then, the sample and blank absorbances were read at 450 nm after a 30 min incubation at room temperature. The absorbance of the blank was subtracted from that of the sample. CUPRAC activity was expressed as equivalents of trolox.

For FRAP reducing power, the sample solution (0.1 mL) was added to premixed a FRAP reagent (2 mL) containing acetate buffer (0.3 M, pH 3.6), 2,4,6-Tris(2-pyridyl)-S-triazine (TPTZ) (10 mM) in 40 mM HCl, and ferric chloride (20 mM) in a ratio of 10:1:1 (v/v/v). Then, the sample absorbance was read at 593 nm after 30 min of incubation at room temperature. FRAP activity was expressed as equivalents of trolox.

For potassium ferricyanide reducing power, the sample solution (0.5 mL) was mixed with a phosphate buffer (0.5 mL, 0.2 M, pH 6.6) and potassium ferricyanide (0.5 mL, 1%), and the mixture was incubated at 50°C for 20 min. Then, trichloroacetic acid (0.5 mL, 10%), deionized water (2.5 mL), and ferric chloride (0.5 mL, 0.1%) were added to this mixture. Finally, the sample absorbance was read at 700 nm. Iron (III) to iron (II) reduction activity was expressed as equivalents of trolox.

For 2,2-azino-bis (3-ethylbenzothiazloine-6-sulfonic acid) (ABTS) radical cation scavenging activity, briefly, the ABTS^·+^ radical cation was produced directly by reacting 7 mM ABTS solution with 2.45 mM potassium persulfate and allowing the mixture to stand for 12−16 hours in dark at the room temperature. Prior to beginning the assay, the ABTS solution was diluted with methanol to an absorbance of 0.700 ± 0.02 at 734 nm. The sample solution (1 mL) was added to ABTS solution (2 mL) and mixed. The sample absorbance was read at 734 nm after 30 min of incubation at room temperature. The ABTS radical cation scavenging activity was expressed as equivalents of trolox.

For superoxide anion radical scavenging activity, the sample solution (0.25 mL) was added to reaction mixture containing riboflavin (0.1 mL, 0.1 mg/mL), EDTA (0.1 mL, 12 mM), NBT (0.05 mL, 1 mg/mL), phosphate buffer (1 mL, 50 mM, pH 7.8), and 1-butanol (0.5 mL). The reaction mixture was illuminated for 10 min at room temperature and the sample absorbance was read at 560 nm. The unilluminated reaction mixture was used as a blank. The absorbance of the blank was subtracted from that of the sample, and superoxide radical scavenging activity was expressed as equivalents of trolox.

For nitric oxide (.NO) radical scavenging activity, the sample solution (0.5 mL) was mixed with sodium nitroprusside (0.5 mL, 5 mM) in phosphate buffer (0.2 M, pH 7.4) and incubated for 150 min at room temperature. Similarly, a blank was prepared by adding sample solution (0.5 mL) to a phosphate buffer (0.5 mL). Diluted Griess reagent (1 mL, 1:1) was added to the incubated sample and allowed to stand for 30 min. The sample and blank absorbances were read at 548 nm. The absorbance of the blank was subtracted from that of the sample, and the nitric oxide radical scavenging activity was expressed as equivalents of trolox.

### Enzyme Inhibitory Activity

The enzymes inhibitory activities (acetylcholinesterase (AChE), butyrylcholinesterase (BChE), tyrosinase, α-glucosidase, and α-amylase) were evaluated by using the methods reported elsewhere (Sarikurkcu et al., 2015).

Cholinesterase (ChE) inhibitory activity was measured using Ellman's method. The sample solution (50 μL) was mixed with DTNB (0.3 mM, 125 μL) and AChE (or BuChE, 0.026 unit/mL) solution (25 μL) in a Tris–HCl buffer (50 mM, pH 8.0) in a 96-well microplate and incubated for 15 min at 25°C. The reaction was then initiated with the addition of acetylthiocholine iodide (ATCI) or butyrylthiocholine chloride (BTCl) (1.5 mM, 25 μL). Similarly, a blank was prepared by adding sample solution to all reaction reagents without enzyme (AChE or BuChE) solution. The sample and blank absorbances were read at 405 nm after a 10 min incubation at 25°C. The absorbance of the blank was subtracted from that of the sample and the cholinesterase inhibitory activity was expressed as equivalents of galantamine. The values of the calibration curves: for AChE, absorbance = 2.104[µg galantamine] – 0.0033 (R^2^: 0.9992); for BChE, absorbance = 1.965[µg galantamine] + 0.0012 (R^2^: 0.9999)

Tyrosinase inhibitory activity was measured using the modified dopachrome method with L-DOPA as substrate. The sample solution (25 μL) was mixed with tyrosinase solution (200 unit/mL, 40 μL) and phosphate buffer (100 μL, pH 6.8) in a 96-well microplate and incubated for 15 min at 25°C. The reaction was then initiated with the addition of L-DOPA (10 mM, 40 μL). Similarly, a blank was prepared by adding sample solution to all reaction reagents without enzyme (tyrosinase) solution. The sample and blank absorbances were read at 492 nm after a 10 min incubation at 25°C. The absorbance of the blank was subtracted from that of the sample, and the tyrosinase inhibitory activity was expressed as equivalents of kojic acid. The value of the calibration curve: absorbance = 1.5235[mg kojic acid] + 0.0055 (R^2^: 0.9981).

α-Amylase inhibitory activity was performed using Caraway–Somogyi iodine/potassium iodide (IKI) method. The sample solution (25 μL) was mixed with α-amylase solution (50 μL) in a phosphate buffer (pH 6.9 with 6 mM sodium chloride) in a 96-well microplate and incubated for 10 min at 37°C. After pre-incubation, the reaction was initiated with the addition of starch solution (50 μL, 0.05%). Similarly, a blank was prepared by adding sample solution to all reaction reagents without an enzyme (α-amylase) solution. The reaction mixture was incubated 10 min at 37°C. The reaction was then stopped with the addition of HCl (25 μL, 1 M). This was followed by addition of the iodine-potassium iodide solution (100 μL). The sample and blank absorbances were read at 630 nm. The absorbance of the blank was subtracted from that of the sample and the α-amylase inhibitory activity was expressed as equivalents of acarbose. The value of the calibration curve: absorbance = 2.9996[mg acarbose] + 0.006 (R^2^: 0.9952)

For α-glucosidase inhibitory activity, the sample solution (50 μL) was mixed with glutathione (1 mg/mL, 50 μL), α-glucosidase solution (0.2 unit/mL, 50 μL) in a phosphate buffer (pH 6.8) and PNPG (10 mM, 50 μL) in a 96-well microplate and incubated for 15 min at 37°C. Similarly, a blank was prepared by adding sample solution to all reaction reagents without enzyme (α-glucosidase) solution. The reaction was then stopped with the addition of sodium carbonate (50 μL, 0.2 M). The sample and blank absorbances were read at 400 nm. The absorbance of the blank was subtracted from that of the sample and the α-glucosidase inhibitory activity was expressed as equivalents of acarbose. The value of the calibration curve: absorbance = 8.704[mg acarbose] + 0.0157 (R^2^: 0.9961). In **Supplementary Material**, **Figure S1–S5** were reported the calibration curves used for enzyme inhibitory activity.

### Statistical Analysis

All of the assays were carried out in triplicate. Results obtained from all the assays were presented as mean and standard deviation values (mean ± SD). ANOVA (one-way analysis of variance) and Tukey's honestly significant difference *post hoc* test with α = 0.05 (SPSS v. 22.0) were used to determine the differences between the assays. The Pearson linear correlation was used to evaluate the association between the results.

## Results and Discussion

### Determination of the Total Phenolic, Flavonoid, and Condensed Tannin Content and Quantitative LC–ESI–MS/MS Analysis

In order to obtain a possible correlation between biological activities and bioactive compounds present in the extracts, it is necessary to evaluate the total phenolic, flavonoid, and condensed tannin content. This information, even if not completely useful for the plant extract characterization, allows us to better highlight that some of the biological activities could be related not only to the single compounds but also to the phytocomplex. The results are presented in **Table 2**.

**Table 2:** Total phenolics, flavonoids, and condensed tannins of *S. perfoliata* extracts.^x^

Samples	Total phenolics (mg GAEs/g extract)	Total flavonoids (mg QEs/g extract)	Total condensed tannins (mg CEs/g extract)
Ethyl acetate	36.68 ± 0.38^c^	21.96 ± 0.25^c^	7.41 ± 0.04^b^
Methanol	41.64 ± 0.99^b^	40.90 ± 1.80^a^	10.65 ± 0.28^a^
Water	52.18 ± 0.75^a^	29.13 ± 0.26^b^	10.76 ± 0.05^a^

^x^Different subscripts in the same column indicate significant difference (by ANOVA, p < 0.05). GAEs, QEs, and CEs, gallic acid, quercetin, and catechin equivalents, respectively.

The spectrophotometric assays used herein (Zengin et al., 2014) allowed us to spot that water extracts were richest in terms of phenolics and condensed tannins (52.18 mg GAEs/g extract and 10.76 mg CEs/g extract, respectively), while methanol extracts were the richest in terms of flavonoids (40.90 mg QEs/g extract). Ethyl acetate extracts showed the lowest quantities for phenolics, flavonoids, and condensed tannins.

**Figure 1:**
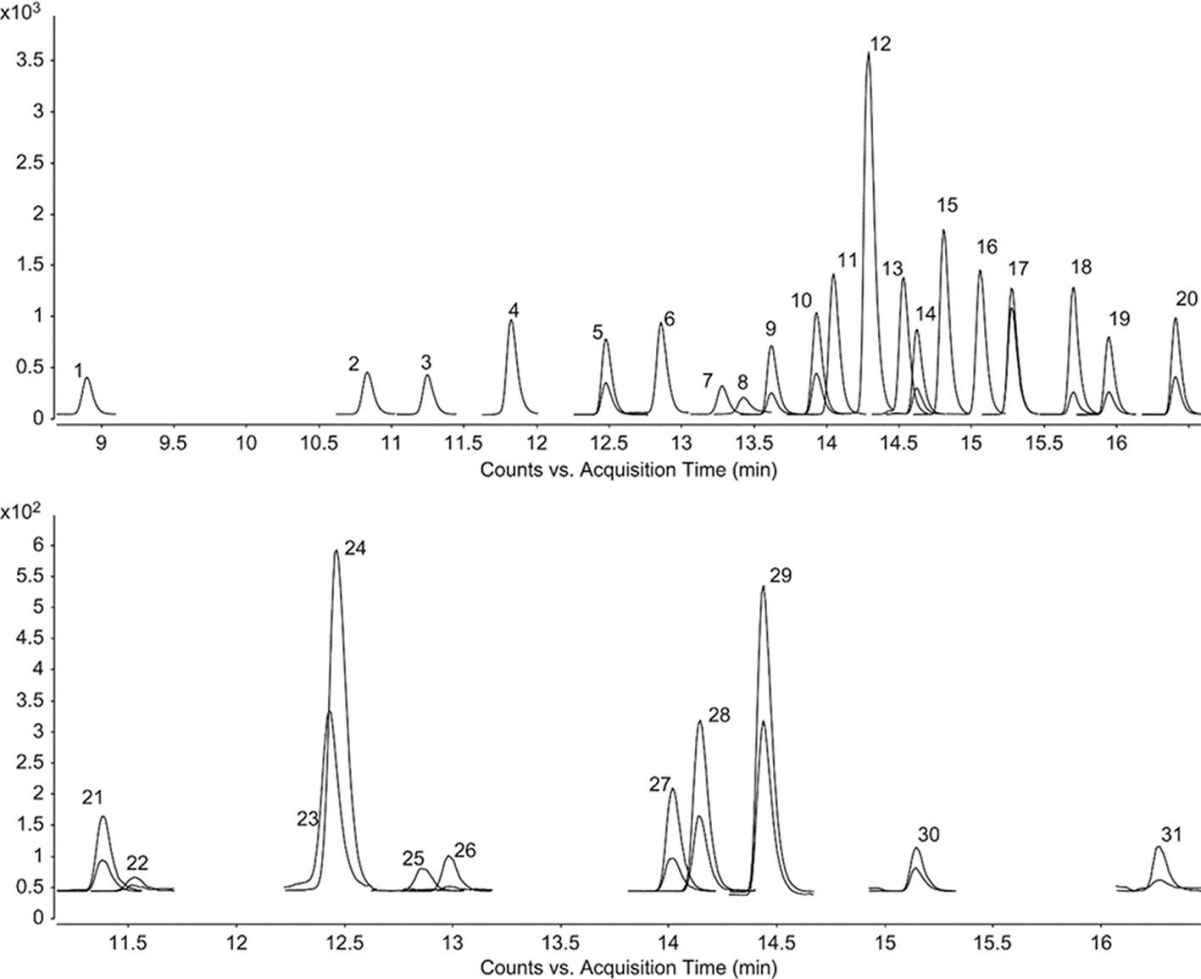
LC–ESI–MS/MS MRM chromatograms of phenolic compounds. 1–31 represent the chromatograms of gallic acid, protocatechuic acid, 3,4-dihydroxyphenylacetic acid, chlorogenic acid, (−)-epicatechin, caffeic acid, 3-hydroxybenzoic acid, vanillin, verbascoside, taxifolin, p-coumaric acid, luteolin 7-glucoside, hyperoside, rosmarinic acid, apigenin 7-glucoside, 2-hydroxycinnamic acid, eriodictyol, quercetin, luteolin, apigenin, (+)-catechin, pyrocatechol, 2,5-dihydroxybenzoic acid, 4-hydroxybenzoic acid, vanillic acid, syringic acid, sinapic acid, ferulic acid, hesperidin, pinoresinol, and kaempferol, respectively. The phenolic concentrations are 400 μg L^-1^.

The same trend could also be observed when a deep investigation was carried out using a more complex instrument configuration. Using LC-ESI-MS/MS instrument configuration it is possible to obtain, for the considered analytes, the chemical profiles for the tested extracts, and **Figure 1** displays a chromatogram showing the resolution of the chemical standards considered in this study (Cittan and Çelik, 2018). The results are presented in **Table 3**.

**Table 3:** Amounts (µg/g extract) of selected phytochemicals in the tested extracts.^x^

Class	Compounds	Ethyl acetate	Methanol	Water
Hydroxzbenzoic acids	Gallic acid	0.54 ± 0.03^b^	11.26 ± 0.12^a^	nd^y^
	Protocatechuic acid	0.95 ± 0.01^c^	54.28 ± 1.58^b^	141.83 ± 0.25^a^
	3,4-Dihydroxyphenylacetic acid	nd	1.39 ± 0.08^b^	13.11 ± 0.50^a^
	2,5-Dihydroxybenzoic acid	nd	4.97 ± 0.05	nd
	4-Hydroxybenzoic acid	1.11 ± 0.03^c^	25.74 ± 0.09^b^	143.00 ± 0.83^a^
	Vanillic acid	2.15 ± 0.28^c^	21.29 ± 0.43^b^	59.58 ± 1.38^a^
	Syringic acid	nd	8.12 ± 0.76^b^	27.92 ± 0.98^a^
Hydroxycinnamic acids	Chlorogenic acid	10.98 ± 0.43^c^	9975.82 ± 323.48^b^	24933.41 ± 1028.49^a^
	Caffeic acid	0.53 ± 0.01^c^	67.15 ± 0.27^b^	186.83 ± 2.91^a^
	Ferulic acid	1.12 ± 0.02^c^	19.29 ± 0.03^b^	61.84 ± 0.55^a^
	Rosmarinic acid	1.52 ± 0.19	nd	nd
	Sinapic acid	nd	0.70 ± 0.12	nd
	*p*-Coumaric acid	nd	14.15 ± 0.19^b^	22.48 ± 0.17^a^
Flavonoids	Luteolin 7-glucoside	nd	2.45 ± 0.06^b^	6.23 ± 0.27^a^
	Hesperidin	nd	0.82 ± 0.09	nd
	Hyperoside	nd	2.20 ± 0.25^b^	5.12 ± 0.13^a^
	Apigenin 7-glucoside	nd	278.44 ± 14.58^b^	1437.53 ± 316.28^a^
	Apigenin	1.51 ± 0.03^c^	29.64 ± 0.13^b^	35.41 ± 0.90^a^
	(-)-Epicatechin	nd	0.89 ± 0.14	nd
Others	Verbascoside	136.79 ± 26.83^c^	29033.77 ± 145.61^b^	50951.10 ± 1175.41^a^
	Pinoresinol	nd	4.96 ± 0.29^b^	12.18 ± 2.32^a^

^x^Different subscripts in the same row indicate significant difference (p < 0.05); ^y^ nd, not detected. Unreported analytes respect to the method are absent (nd) in all analyzed samples. LOD and LOQ, limit of detection and limit of quantification, respectively.

**Figure 2:**
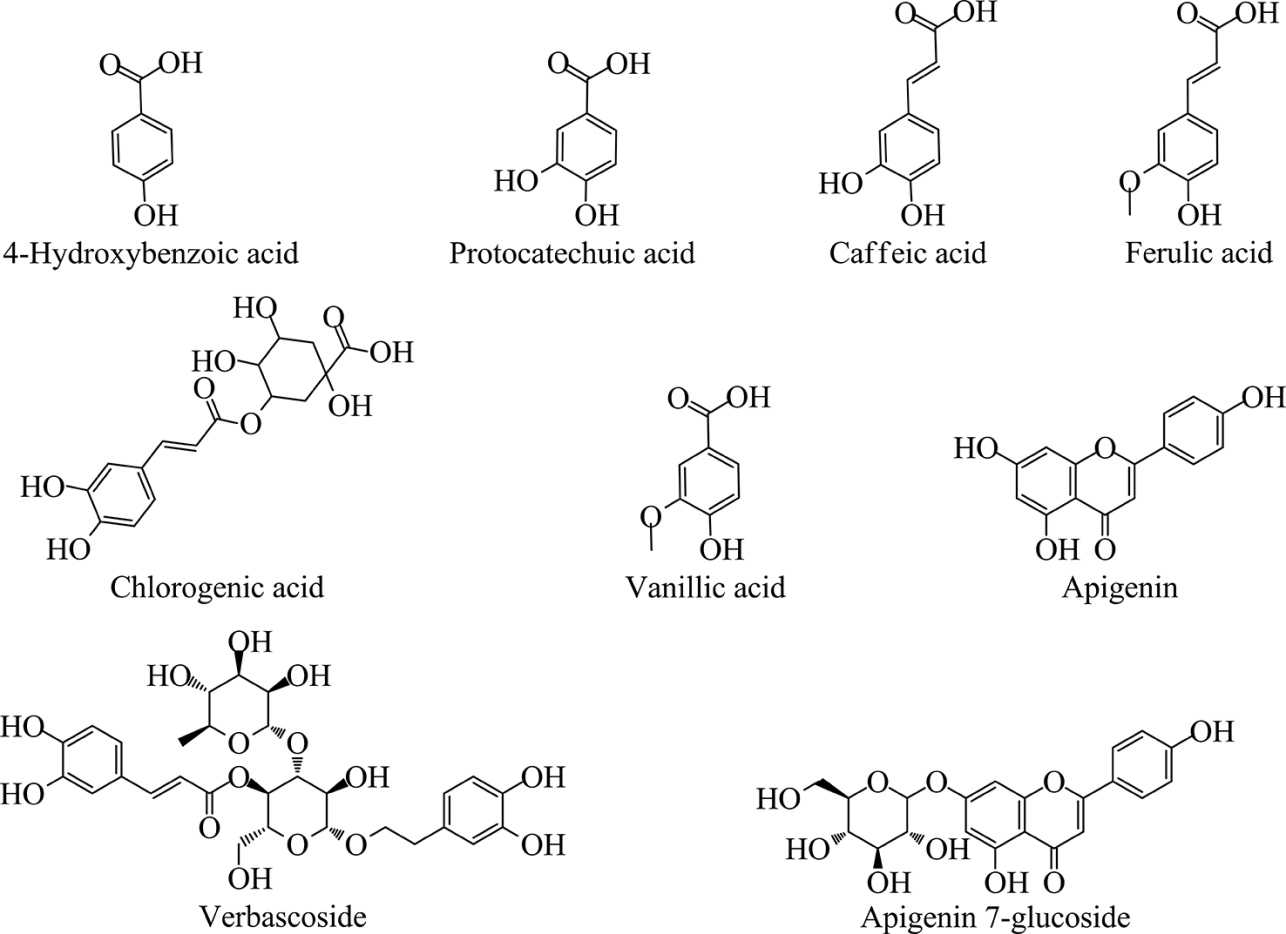
Chemical structures of the major analytes quantified by LC-ESI-MS/MS method.

In **Table 3**, the amounts (expressed as μg/g dry extract) show that water extracts were richest with respect to methanol and ethyl acetate extracts. **Figure 2** shows the most abundant compounds quantified in the LC-ESI-MS/MS analyses.

These analyses highlighted that *Sideritis perfoliata* contains mainly verbascoside, chlorogenic acid, and apigenin 7-glucoside (50951.10, 24933.41, and 1437.53 μg/g dry extract, respectively). Interestingly, the methanol extract showed a more complete phytochemical pattern in comparison with the other extracts, even if the values were lower as a total amount (39557.33 μg/g methanol dry extract against 78037.37 μg/g water dry extract). Verbascoside represents 65.3% of the total amount for the water extract, 73.4% for methanol extract, and 87% of the ethyl acetate extract. These results are in accordance with the previous works reported in literature (Küpeli et al., 2007; Petreska et al., 2011).

 Chlorogenic acid is the second most abundant compound present in all three extracts. This compound was confirmed also in *Sideritis raeseri* (Samanidou et al., 2012). In the same work, apigenin was also found after aglycones hydrolysis at 1.2 μg/g amount.

Furthermore, information on gallic acid, (-)-epicatechin, syringic acid, *p*-coumaric acid, ferulic acid, and luteolin 7-glucoside presence are available in the works of Proestos and Komaitis (2013). This work confirmed the presence of these compounds in the *Sideritis cretica* extracts obtained by extraction with 62.5% aqueous methanol.

To the best of our knowledge, no papers reports the presence of protocatecheuic acid, 4-hydroxybenzoic acid, vanillic acid, and caffeic acid reported herein in all the three extracts.

### Biological Activities

In order to evaluate the biological activities, the different *Sideritis perfoliata* extracts were submitted to biological assays: total antioxidant (by phosphomolybdenum and β-carotene-linoleic acid), ferrous ion chelating, reducing power [cupric ion reducing (CUPRAC), ferric reducing antioxidant power (FRAP), and potassium ferricyanide], and free radical scavenging [on 1,1-diphenyl-2-picrylhydrazyl (DPPH^·^) radical, 2,2-azino-bis (3-ethylbenzothiazloine-6-sulphonic acid) radical cation (ABTS^·+^), superoxide anion (O_2_^·-^) radical, and nitric oxide (^·^NO) radical]. The results are presented in **Table 4**.

**Table 4:** Radical scavenging, reducing power, ferrous ion chelating, and total antioxidant (by phosphomolybdenum and β-carotene bleaching methods) activities of *S. perfoliata* extracts.^x^

	Radical scavenging activity (mg TEs/g extract)				Reducing power (mg TEs/g extract)			β-carotene bleaching (%)^y^	Phosphomolybdenum (mg AAEs/g extracts)	Ferrous ion chelating (mg EDTAEs/g extracts)
	DPPH	ABTS	Superoxide	Nitric oxide	Potassium ferricyanide	CUPRAC	FRAP			
Ethyl acetate	101.61 ± 0.64^c^	89.47 ± 6.58^b^	39.74 ± 3.02^c^	42.15 ± 12.86^b^	109.14 ± 2.02^b^	92.41 ± 5.93^b^	58.79 ± 1.75^c^	22.32 ± 0.59^e^	87.21 ± 6.70^c^	3.01 ± 0.91^b^
Methanol	266.25 ± 1.07^b^	136.53 ± 0.01^a^	150.00 ± 0.60^b^	205.37 ± 15.78^a^	219.57 ± 0.59^a^	197.89 ± 14.79^a^	137.95 ± 0.07^b^	17.31 ± 0.62^f^	140.09 ± 5.38^b^	6.21 ± 0.84^b^
Water	405.53 ± 8.59^a^	149.71 ± 3.29^a^	159.62 ± 2.12^a^	209.09 ± 12.86^a^	225.88 ± 0.42^a^	210.69 ± 5.47^a^	161.19 ± 0.96^a^	49.58 ± 0.34^d^	222.39 ± 15.34^a^	40.79 ± 2.96^a^
BHA	–	–	–	–	–	–	–	80.30 ± 0.61^b^	–	–
BHT	–	–	–	–	–	–	–	76.67 ± 0.41^c^	–	–
Trolox	–	–	–	–	–	–	–	85.58 ± 0.04^a^	–	–

^x^Different subscripts in the same row indicate significant difference (by ANOVA, p < 0.05). TEs, AAEs, and EDTAEs, trolox, ascorbic acid, and ethylenediaminetetraacetic acid (disodium salt) equivalents, respectively. ^y^ Given as percentage of % inhibition of the linoleic acid at 2 mg/ml concentration.

Due to the presence of different phytochemicals and related to their highest amounts in the water extract, the biological activities can be ordered as follow: water > methanol > ethyl acetate. Only for β-carotene bleaching assay, the order was water > ethyl acetate > methanol. This could be related to the presence, in ethyl acetate extracts, of the rosmarinic acid. This fact could obviate to a lower phytochemical amounts with respect the other extracts. Additionally, the correlation of rosmarinic acid and the β-carotene bleaching activity was just reported in literature (Öztürk, 2015).

In the literature, we observed several studies on the members of *Sideritis,* and the species generally indicated significant antioxidant properties. In a recent paper, the cultivated *S. perfoliata* subsp. *perfoliata* was examined in terms of chemical profile and biological abilities (Chrysargyris et al., 2019). In their study, the extracts had considerable antioxidant properties (4.34–13.25 mg TE/g fresh weight in DPPH, 2.61–6.87 mg TE/g fresh weight in ABTS, and 6.89–27.31 mg TE/g fresh weight in FRAP). Also, the authors reported a strong correlation between total phenolic content and these antioxidant abilities. Zengin et al. (2019) reported the antioxidant abilities of *S. ozturkii*, which were 32.48–137.30 mg TE/g extract in DPPH, 46.78–176.45 mg TE/g extract in ABTS, and 132.47–428.61 mg TE/g extract in CUPRAC. Similar to our study, the methanol and water extracts for *S. ozturkii* had stronger abilities than the ethyl acetate extract. This fact was also confirmed by several researchers (Zengin et al., 2014; Sagir et al., 2017; Celep et al., 2019). As can be seen in literature, several researchers reported antioxidant parameters of some *Sideritis* species as IC_50_ or EC_50_ values. At this point, we could not compare with these studies. Taken together, the *Sideritis* genus has great interest in terms of good sources of natural antioxidants to combat oxidative stress.

### Enzyme Inhibitory Activity

Enzyme inhibition theory is considered one of effective therapeutic approaches to control global health problems, including Alzheimer's disease and diabetes mellitus. In this context, the inhibitory activities of tested extracts were tested against cholinesterases (AChE and BChE), α-amylase, α-glucosidase, and tyrosinase. The results are presented in **Table 5**. The inhibitory activities against all enzymes ranged according to the extraction solvents. Generally, the water extract demonstrated lower activity against all enzymes except for tyrosinase. The observed tyrosinase inhibitory ability for water extracts may be linked to higher levels of phenolics. As is consistent with this approach, a positive correlation was observed between the antityrosinase effect and total phenolics (**Table 5**). Similar findings were also supported by several researchers (Kim and Uyama, 2005; Taofiq et al., 2017). The ethyl acetate and methanol extracts showed similar inhibitory effects against AChE, tyrosinase, and α-glucosidase.

**Table 5:** Enzyme inhibitory activities of *S. perfoliata* extracts.^x^

	AChE (mg GALAEs/g extract)^y^	BChE (mg GALAEs/g extract)^y^	Tyrosinase (mg KAEs/g extract)^z^	α-Amylase (mg ACEs/g extract)^w^	α-Glucosidase (mg ACEs/g extract)^w^
Ethyl acetate	0.23 ± 0.03^ab^	0.53 ± 0.01^a^	5.97 ± 0.76^b^	36.57 ± 1.92^b^	175.90 ± 1.46^a^
Methanol	0.26 ± 0.03^a^	0.35 ± 0.03^b^	7.80 ± 0.01^b^	48.76 ± 2.19^a^	169.12 ± 4.22^a^
Water	0.14 ± 0.03^b^	0.09 ± 0.01^c^	14.65 ± 0.16^a^	33.28 ± 0.01^b^	112.94 ± 5.69^b^

^x^Different subscripts in the same row indicate significant difference (by ANOVA, p < 0.05). GALAEs, KAEs, and ACEs, galanthamine, kojic acid, and acarbose equivalents, respectively.

Correlation coefficients between the assays are reported in **Table 6**. The aim of this Table is to highlight the possible correlation between the results obtained using different tests. Interestingly, this correlation could bring to a positive or negative coefficient, and in several cases these values are higher than 0.9, highlighting that the applied assays could be used independently for the determination of a specific activities.

**Table 6:** Correlation coefficients between the assays.^x^

	TACB	TAP	MCA	DPPH	ABTS	SAR	NOR	PFRP	CUPRAC	FRAP	ACIA	BCIA	TIA	AAIA	AGIA
Total condensed tannins	0.397	0.814	0.588	0.902	0.984	0.999^y^	0.999^z^	0.999^z^	0.998^y^	0.982	-0.305	-0.826	0.684	0.287	-0.606
Total flavonoids	-0.280	0.256	-0.063	0.419	0.643	0.742	0.776	0.758	0.724	0.636	0.373	-0.276	0.062	0.832	0.041
Total phenolics	0.895	0.997	0.971	0.968	0.868	0.792	0.759	0.777	0.808	0.873	-0.847	-0.995	0.993	-0.397	-0.976
Protocatechuic acid	0.863	0.999^z^	0.953	0.982	0.899	0.831	0.800	0.817	0.845	0.903	-0.810	-0.999^y^	0.983	-0.336	-0.959
Chlorogenic acid	0.851	0.999^z^	0.945	0.987	0.909	0.844	0.814	0.831	0.858	0.913	-0.795	-0.999^z^	0.979	-0.313	-0.952
4-Hydroxybenzoic acid	0.953	0.972	0.996	0.918	0.781	0.688	0.649	0.670	0.707	0.786	-0.919	-0.967	0.999^y^	-0.534	-0.998^y^
Vanillic acid	0.888	0.998^y^	0.967	0.972	0.876	0.801	0.769	0.787	0.817	0.880	-0.839	-0.996	0.991	-0.383	-0.972
Caffeic acid	0.875	0.999^y^	0.960	0.978	0.888	0.817	0.786	0.803	0.832	0.892	-0.824	-0.998^y^	0.987	-0.358	-0.966
Verbascoside	0.733	0.979	0.865	0.999^y^	0.973	0.932	0.911	0.922	0.941	0.975	-0.664	-0.983	0.921	-0.124	-0.875
Ferulic acid	0.905	0.995	0.976	0.962	0.857	0.778	0.744	0.763	0.795	0.861	-0.859	-0.992	0.996	-0.418	-0.981
Apigenin 7-glucoside	0.947	0.977	0.994	0.926	0.794	0.703	0.664	0.686	0.722	0.799	-0.911	-0.972	0.999^y^	-0.517	-0.996
Apigenin	0.513	0.883	0.689	0.951	0.999^y^	0.996	0.990	0.994	0.998^y^	0.998^y^	-0.427	-0.892	0.774	0.160	-0.704

^x^Data represent Pearson Correlation Coefﬁcient *R*. (TACB, total antioxidant activity by β-carotene bleaching method; TAP, total antioxidant activity by phosphomolybdenum method; MCA, metal chelating activity; DPPH, DPPH radical scavenging activity; ABTS, ABTS radical scavenging activity; SAR, superoxide anion radical scavenging activity; NOR, nitric oxide radical scavenging activity; PFRP, potassium ferricyanide reducing power potential; CUPRAC, CUPRAC reducing power potential; FRAP, FRAP reducing power potential; ACIA, acetyl cholinesterase inhibition activity; BCIA, butyrylcholinesterase inhibitory activity; TIA, tyrosinase inhibitory activity; AAIA, α-amylase inhibition activity; AGIA, α-glucosidase inhibition activity); ^y^ indicates p < 0.05; ^z^ indicates p < 0.01.

Similar to antioxidant properties of the members of the genus *Sideritis*, some studies were performed on enzyme inhibitory effects of *Sideritis* species. Generally, the genus *Sideritis* exhibited significant enzyme inhibitory potentials. For example, the AChE and tyrosinase inhibitory effects of *S. ozturkii* extracts (Zengin et al., 2019) were higher than those of our presented results. Also, Celep et al. (2019) reported that more than 20% of *S. trojana* extracts had inhibitory effects on α-amylase and α-glucosidase. In an earlier study (Zengin et al., 2014), the inhibitory effects of *S. galatica* extracts and the ethyl acetate and methanol extracts were more active than water, which is consistent with our results. In addition to these studies, several researchers focused on enzyme inhibitory properties of some *Sideritis* essential oils (Zengin et al., 2016; Tadić et al., 2017; Deveci et al., 2019). Altogether, the genus *Sideritis* is a significant source of natural enzyme inhibitors.

### Conclusion

In the present work, different biological effects for three different *Sideritis perfoliata* extracts as well as their chemical profiles obtained using highly hyphenated instrument configuration were reported. From the results reported herein, it was apparent that the biological activities and chemical profiles were strictly dependent on the extraction solvents system. Verbascoside, chlorogenic acid, and apigenin 7-glucoside were the major phenolic components determined in these extracts. Generally, the methanol and water extracts showed greater antioxidant activities respect to ethyl acetate extracts. Investigated *Sideritis perfoliata* showed worthy inhibitory properties with regards to key enzymes associated to Alzheimer's disease and Diabetes mellitus health diseases.

In recent years, novel and safe products have displayed great interest in the prevention and management of global health problems, including Alzheimer's disease, diabetes mellitus, and oxidative stress. In this sense, our findings could contribute significant information for scientific platforms. From the results reported herein, the investigated *Sideritis perfoliata* could be considered as a favorable font of natural agents for the future development of new pharmaceuticals and as a potential source of biological active compounds for the food supplements, such as nutraceuticals, functional foods or biologically-active products.

It is necessary to highlight that further experimental studies, such as *in vivo* models and toxicological assays, are necessary for the studied plant material. Additional information is required also to standardize and to modify the production process to an industrial scale.

## Author Contributions

BK and CS set up and carried out experiments. ML, AM, and GZ carried out the data analysis and paper writing and revision.

## Conflict of Interest

The authors declare that the research was conducted in the absence of any commercial or financial relationships that could be construed as a potential conflict of interest.

The reviewer AU declared a shared affiliation, with no collaboration, with one of the authors, GK, to the handling editor at time of review.

## References

[B1] Barber J. C. (2000). Evolution of endemic Sideritis (Lamiaceae) in Macaronesia: insights from a chloroplast DNA restriction site analysis. Syst. Bot. 25 (4), 633–647. 10.2307/2666725

[B2] Boy H. I. A. Rutilla A. J. H. Santos K. A. Ty A. M. T. Alicia I. Y. Mahboob T. (2018). Recommended medicinal plants as source of natural products: a review. Dig. Chin. Med. 1 (2), 131–142. 10.1016/S2589-3777(19)30018-7

[B3] Celep E. Seven M. Akyüz S. İnan Y. Yesilada E. (2019). Influence of extraction method on enzyme inhibition, phenolic profile and antioxidant capacity of Sideritis trojana Bornm. S. Afr. J. Bot. 121, 360–365. 10.1016/j.sajb.2018.11.026

[B4] Charami M. T. Lazari D. Karioti A. Skaltsa H. Hadjipavlou-Litina D. Souleles C. (2008). Antioxidant and antiinflammatory activities of Sideritis perfoliata subsp. perfoliata (Lamiaceae). Phytother. Res. 22 (4), 450–454. 10.1002/ptr.2333 18386254

[B5] Çelik Í. Ersanlı C. C. Köseoğlu R. Akşit H. Erenler R. Demirtaş I. (2016). Crystal structure of 3,4a,7,7,10a-penta-methyl-3-vinyl-dodeca-hydro-1H-benzo[f]chromen-9-ol isolated from Sideritis perfoliata. Acta Crystallogr. E Crystallogr. Commun. 72 (Pt 10), 1380–1382. 10.1107/S2056989016013864 27746923 PMC5050758

[B6] Chrysargyris A. Kloukina C. Vassiliou R. Tomou E. M. Skaltsa H. Tzortzakis N. (2019). Cultivation strategy to improve chemical profile and anti-oxidant activity of Sideritis perfoliata L. subsp. perfoliata. Ind. Crop Prod. 140, 111694. 10.1016/j.indcrop.2019.111694

[B7] Cittan M. Çelik A. (2018). Development and validation of an analytical methodology based on liquid chromatography–electrospray tandem mass spectrometry for the simultaneous determination of phenolic compounds in olive leaf extract. J. Chromatogr. Sci. 56 (4), 336–343. 10.1093/chromsci/bmy003 29373655

[B8] P. H. Davis (Ed.). (1982). Flora of Turkey and the East Aegean Islands Vol. 7 (Edinburgh: University Press).

[B9] Deveci E. Tel-Çayan G. Usluer Ö. Duru M. E. (2019). Chemical composition, antioxidant, anticholinesterase and anti-tyrosinase activities of essential oils of two *Sideritis* species from Turkey. Iran J. Pharm. Res. 18 (2), 903. 10.22037/ijpr.2019.1100657 31531072 PMC6706731

[B10] Di Sotto A. Vecchiato M. Abete L. Toniolo C. Giusti A. M. Mannina L. (2018). *Capsicum annuum* L. var. Cornetto di Pontecorvo PDO: Polyphenolic profile and *in vitro* biological activities. J. Funct. Foods 40, 679–691. 10.1016/j.jff.2017.11.041

[B11] González-Burgos E. Carretero M. E. Gómez-Serranillos M. P. (2011). *Sideritis* spp.: Uses, chemical composition and pharmacological activities—A review. J. Ethnopharmacol. 135 (2), 209–225. 10.1016/j.jep.2011.03.014 21420484

[B12] Küpeli E. Sahin F. P. Yeşilada E. Caliş I. Ezer N. (2007). In vivo anti-inflammatory and antinociceptive activity evaluation of phenolic compounds from *Sideritis stricta* . Z Naturforsch. C. 62 (7-8), 519–525. 10.1515/znc-2007-7-810 17913066

[B13] Kim Y. J. Uyama H. (2005). Tyrosinase inhibitors from natural and synthetic sources: structure, inhibition mechanism and perspective for the future. Cell. Mol. Life Sci. CMLS 62 (15), 1707–1723. 10.1007/s00018-005-5054-y 15968468 PMC11139184

[B14] Lall N. Chrysargyris A. Lambrechts I. Fibrich B. Blom Van Staden A. Twilley D. (2019). *Sideritis Perfoliata* (Subsp. Perfoliata) nutritive value and its potential medicinal properties. Antioxidants 8 (11), 521. 10.3390/antiox8110521 PMC6912803 31671566

[B15] Lindqvist C. Albert V. A. (2002). Origin of the Hawaiian endemic mints within North American *Stachys* (Lamiaceae). Am. J. Bot. 89 (10), 1709–1724. 10.3732/ajb.89.10.1709 21665597

[B16] Locatelli C. Melucci D. Locatelli M. (2014). Toxic metals in herbal medicines. A review. Curr. Bioact. Comp. 10 (3), 181–188. 10.2174/1573407210666140716164321

[B17] Locatelli M. Zengin G. Uysal A. Carradori S. De Luca E. Bellagamba G. (2017). Multicomponent pattern and biological activities of seven *Asphodeline* taxa: Potential sources of natural-functional ingredients for bioactive formulations. J. Enz. Inhib. Med. Chem. 32 (1), 60–67. 10.1080/14756366.2016.1235041 PMC6010134 27774819

[B18] Loizzo M. R. Tundis R. Menichini F. Saab A. M. Statti G. A. Menichini F. (2007). Cytotoxic activity of essential oils from labiatae and lauraceae families against *in vitro* human tumor models. Anticancer Res. 27 (5A), 3293–3299. 17970073

[B19] Loizzo M. R. Saab A. M. Tundis R. Menichini F. Bonesi M. Piccolo V. (2008). In vitro inhibitory activities of plants used in Lebanon traditional medicine against angiotensin converting enzyme (ACE) and digestive enzymes related to diabetes. J. Ethnopharmacol. 119 (1), 109–116. 10.1016/j.jep.2008.06.003 18601990

[B20] Melucci D. Locatelli M. Locatelli C. (2013). Trace level voltammetric determination of heavy metals and total mercury in tea matrices (*Camellia sinensis*) doi: 10.1016/j.fct.2013.10.029. Food Chem. Toxicol. 62, 901–907. 10.1016/j.fct.2013.10.029 24416778

[B21] Melucci D. Locatelli M. De Laurentiis F. Zengin G. Locatelli C. (2018). Herbal medicines: application of a sequential voltammetric procedure to the determination of mercury, copper, lead, cadmium and zinc at trace level. Lett. Drug Des. Discovery 15 (3), 270–280. 10.2174/1570180814666170412124634

[B22] Öztürk N. (2015). Phenolic composition and antioxidant activity of the different extracts from *Thymus longicaulis* C Presl. subsp. *longicaulis* var. *longicaulis* and *T. longicaulis* C. Presl. subsp. *longicaulis* var. *subisophyllus* growing in Turkey. Pak. J. Pharm. Sci. 28 (2), 465–472. 25730781

[B23] Petreska J. Stefkov G. Kulevanova S. Alipieva K. Bankova V. Stefova M. (2011). Phenolic compounds of mountain tea from the Balkans: LC/DAD/ESI/MSn profile and content. Nat. Prod. Commun. 6 (1), 21–30. 10.1177/1934578X1100600107 21366039

[B24] Proestos C. Komaitis M. (2013). Analysis of naturally occurring phenolic compounds in aromatic plants by RP-HPLC coupled to diode array detector (DAD) and GC-MS after Silylation. Foods 2, 90–99. 10.3390/foods2010090 28239100 PMC5302235

[B25] Sagir Z. O. Carikci S. Kilic T. Goren A. C. (2017). Metabolic profile and biological activity of Sideritis brevibracteata PH Davis endemic to Turkey. Int. J. Food Prop. 20 (12), 2994–3005. 10.1080/10942912.2016.1265981

[B26] Samanidou V. Tsagiannidis A. Sarakatsianos I. (2012). Simultaneous determination of polyphenols and major purine alkaloids in Greek *Sideritis* species, herbal extracts, green tea, black tea, and coffee by high-performance liquid chromatography-diode array detection. J. Sep. Sci. 35 (4), 608–615. 10.1002/jssc.201100894 22282422

[B27] Sarikurkcu C. Uren M. C. Tepe B. Cengiz M. Kocak M. S. (2014). Phenolic content, enzyme inhibitory and antioxidative activity potentials of *Phlomis nissolii* and *P. pungens* var. *pungens* . Ind. Crops Prod. 62, 333–340. 10.1016/j.indcrop.2014.09.002

[B28] Sarikurkcu C. Zengin G. Oskay M. Uysal S. Ceylan R. Aktumsek A. (2015). Composition, antioxidant, antimicrobial and enzyme inhibition activities of two *Origanum vulgare* subspecies (subsp *vulgare* and subsp *hirtum*) essential oils. Ind. Crops Prod. 70, 178–184. 10.1016/j.indcrop.2015.03.030

[B29] Shen B. (2015). A new golden age of natural products drug discovery. Cell 163 (6), 1297–1300. 10.1016/j.cell.2015.11.031 26638061 PMC5070666

[B30] Sobolev A. P. Mannina L. Capitani D. Sanzò G. Ingallina C. Botta B. (2018). A multi-methodological approach in the study of Italian PDO “Cornetto di Pontecorvo” red sweet pepper. Food Chem. 255, 120–131. 10.1016/j.foodchem.2018.02.050 29571457

[B31] Tadić V. Jeremic I. Dobric S. Isakovic A. Markovic I. Trajkovic V. (2012). Anti-inflammatory, gastroprotective, and cytotoxic effects of *Sideritis scardica* extracts. Planta Med. 78 (5), 415–427. 10.1055/s-0031-1298172 22274814

[B32] Tadić V. Oliva A. Božović M. Cipolla A. De Angelis M. Vullo V. (2017). Chemical and antimicrobial analyses of *Sideritis roman*a L. subsp. *purpurea* (Tal. ex Benth.) Heywood, an endemic of the Western Balkan. Molecules 22 (9), 1395. 10.3390/molecules22091395 PMC6151398 28832536

[B33] Taofiq O. González-Paramás A. M. Barreiro M. F. Ferreira I. C. (2017). Hydroxycinnamic acids and their derivatives: Cosmeceutical significance, challenges and future perspectives, a Review. Molecules 22 (2), 1–24. 10.3390/molecules22020281 PMC6155946 28208818

[B34] Uysal S. Zengin G. Locatelli M. Bahadori M. B. Mocan A. Bellagamba G. (2017). Cytotoxic and enzyme inhibitory potential of two *Potentilla* species (*P. speciosa* L. and *P. reptans* Willd.) and their chemical composition. Front. Pharmacol. 8 (290), 1–11. 10.3389/fphar.2017.00290 28588492 PMC5441381

[B35] Villar A. Recio M. C. Ríos J. L. Zafra-Polo M. C. (1986). Antimicrobial activity of essential oils from *Sideritis* species. Die Pharm. 41 (4), 298–299. 3523549

[B36] Zengin G. Sarikurkcu C. Aktumsek A. Ceylan R. (2014). Sideritis galatica Bornm.: a source of multifunctional agents for the management of oxidative damage, Alzheimer's's and diabetes mellitus. J. Funct. Food. 11, 538–547. 10.1016/j.jff.2014.08.011

[B37] Zengin G. Sarikurkcu C. Aktumsek A. Ceylan R. Ceylan O. (2014). A comprehensive study on phytochemical characterization of *Haplophyllum myrtifolium* Boiss. endemic to Turkey and its inhibitory potential against key enzymes involved in Alzheimer, skin diseases and type II diabetes. Ind. Crops Prod. 53, 244–251. 10.1016/j.indcrop.2013.12.043

[B38] Zengin G. Sarikurkcu C. Uyar P. Aktumsek A. Uysal S. Kocak M. S. (2015). *Crepis foetida* L. subsp *rhoeadifolia* (Bleb.) Celak. as a source of multifunctional agents: Cytotoxic and phytochemical evaluation. J. Funct. Foods 17, 698–708. 10.1016/j.jff.2015.06.041

[B39] Zengin G. Sarıkürkçü C. Aktümsek A. Ceylan R. (2016). antioxidant potential and inhibition of key enzymes linked to alzheimer's diseases and diabetes mellitus by monoterpene-rich essential oil from *sideritis galatica* bornm. endemic to turkey. Rec. Nat. Prod. 10 (2), 195–206.

[B40] Zengin G. Uğurlu A. Baloglu M. C. Diuzheva A. Jekő J. Cziáky Z. (2019). Chemical fingerprints, antioxidant, enzyme inhibitory, and cell assays of three extracts obtained from *Sideritis ozturkii* Aytaç & aksoy: an endemic plant from turkey. J. Pharm. Biomed. Anal. 171, 118–125. 10.1016/j.jpba.2019.04.011 30986761

