# Supplementary material for: Phenolic Profile and Bioactivities of Sideritis perfoliata L.: The Plant, Its Most Active Extract, and Its Broad Biological Properties
Source: Front Pharmacol. 2020 Feb 14;10:1642. doi: 10.3389/fphar.2019.01642 (PMC7034418; doi:10.3389/fphar.2019.01642)
Supplement: Supplementary file 1 [file DataSheet_1.docx]

**Phenolic profile and bioactivities of *Sideritis perfoliata* L.: the plant, its most active extract, and its broad biological properties**

**Cengiz Sarikurkcu ^1^, Marcello Locatelli ^2,**^, Andrei Mocan ^3^, Gokhan Zengin ^4^, Bulent Kirkan ^5,*^**

*^1^ Department of Analytical Chemistry; Afyonkarahisar University of Health Sciences; Faculty of Pharmacy; Afyonkarahisar; Turkey*

*^2^ Department of Pharmacy; University of Chieti–Pescara “G. d’Annunzio”; Chieti; Italy*

*^3^ Department of Pharmaceutical Botany; “Iuliu Haţieganu” University of Medicine and Pharmacy; Cluj-Napoca, Romania*

*^4^ Department of Biology; Selcuk University; Science Faculty; Konya, Turkey*

*^5^ Water Institute; Süleyman Demirel University; Isparta; Turkey*

*Corresponding Authors:*

* E-mail: [bulentkirkan32@gmail.com](mailto:bulentkirkan32@gmail.com) (Bulent Kirkan)

**** E-mail: [m.locatelli@unich.it](mailto:m.locatelli@unich.it) (Marcello Locatelli)

**Fig S1.** A standard calibration curve of galanthamine for acetylcholinesterase inhibitory activity

**Fig S2.** A standard calibration curve of galanthamine for butyrylcholinesterase inhibitory activity

**Fig S3.** A standard calibration curve of kojic acid for tyrosinase inhibitory activity

**Fig S4.** A standard calibration curve of acarbose for α-amylase inhibitory activity

**Fig S5.** A standard calibration curve of acarbose for α-glucosidase inhibi
